# Pyroptosis in Osteoblasts: A Novel Hypothesis Underlying the Pathogenesis of Osteoporosis

**DOI:** 10.3389/fendo.2020.548812

**Published:** 2021-01-08

**Authors:** Zhengbo Tao, Jinpeng Wang, Kaicheng Wen, Renqi Yao, Wacili Da, Siming Zhou, Yan Meng, Shui Qiu, Keda Yang, Yue Zhu, Lin Tao

**Affiliations:** ^1^ Department of Orthopaedics, First Affiliated Hospital of China Medical University, Shenyang, China; ^2^ Department of Burn Surgery, Changhai Hospital, the Naval Medical University, Shanghai, China

**Keywords:** pyroptosis, osteoporosis, IL-1β, IL-18, NLRP3

## Abstract

Osteoporosis has become a worldwide disease characterized by a reduction in bone mineral density and the alteration of bone architecture leading to an increased risk of fragility fractures. And an increasing number of studies have indicated that osteoblasts undergo a large number of programmed death events by many different causes in osteoporosis and release NLRP3 and interleukin (e.g., inflammatory factors), which play pivotal roles in contributing to excessive differentiation of osteoclasts and result in exaggerated bone resorption. NLRP3 is activated during pyroptosis and processes the precursors of IL-1β and IL-18 into mature forms, which are released into the extracellular milieu accompanied by cell rupture. All of these compounds are the classical factors of pyroptosis. The cellular effects of pyroptosis are commonly observed in osteoporosis. Although many previous studies have focused on the pathogenesis of these inflammatory factors in osteoporosis, pyroptosis has not been previously evaluated. In this review, pyroptosis is proposed as a novel hypothesis of osteoporosis pathogenesis for the first time, thus providing a new direction for the treatment of osteoporosis in the future.

## Background

Osteoporosis is a systemic bone metabolism disease with characteristics of microstructural deterioration of bone tissue and decreased bone density. Approximately 200 million people are suffering from osteoporosis, and the number is increasing every year. The risk of fractures is greatly increased because of the enhanced bone fragility, especially in the hip, vertebrae, and distal forearm. Approximately 8.9 million fractures are caused by osteoporosis, and it has become one of the main causes of death in elderly individuals (1). Bone is an active tissue that is continually resorbed and formed in a process known as remodeling, which is orchestrated by two main cell types: osteoclasts and osteoblasts. Osteoporosis develops when the rate of bone resorption exceeds that of bone formation, and it is caused by natural aging or other pathological conditions (2). Such conditions include low calcium intake (3), estrogen deficiency in postmenopausal women (4, 5), autoimmune diseases, such as rheumatoid arthritis (RA) (6), and the adverse reactions of some drugs, etc.

Over the last 60 years, two seminal clinical observations showing that the decline of ovarian function at menopause leads to a loss of bone mass and estrogen replacement prevents such loss (7, 8) have led to the development of the traditional estrogen-centric perspective of the pathogenesis of osteoporosis (9). In 1999, Eriksen EF et al. demonstrated that bone remodeling in early postmenopausal women is characterized by progressive osteoclastic hyperactivity *via* dramatic elevations in the number of basic multicellular units (10). However, due to advancements in our understanding of the aging process and the effects of aging and age-related oxidative stress (OS) on bones as well as the serious shortcomings of estrogen-based therapies, aging is presented as a pivotal determinant of the loss of strength and bone mass (9). The most enduring theory of aging proposed by Harman D in 1956 elucidates that OS resulting from an increase in intracellular ROS (reactive oxygen species) is the major determinant of aging and lifespan (11). A series of studies have also indicated that ROS can increase osteoclastogenesis by stimulating the receptor activators of the NF-κB ligand (RANKL) and TNF expression (12–14). As the research on inflammatory disease, including rheumatoid arthritis (6), psoriatic arthritis (15), and Crohn’s disease (16), continues, an array of evidence illustrates that inflammation also increases the loss of bone and risk of fractures, thus emphasizing its skeletal relevance (17, 18). Later in 2005, Weitzmann MN and Pacifici R further explained that certain stimuli of the body lead to an enhancement in the adaptive immune function and consequently upregulates the tumor necrosis factor α (TNF-α) produced by activated T cells, which promotes osteoclast formation and bone resorption (19). Obviously, in the process of osteoporosis, a large number of inflammatory factors and inflammatory signaling pathways are involved, which indicates that there is a certain mechanism between the occurrence of inflammation and osteoporosis that remains to be explored. Pyroptosis, a kind of programmed lytic cell death, is highly correlated to inflammation (20). Compared with apoptosis, pyroptosis, another type of programmed cell death that is non-lytic and usually immune-silent, mediates the development of many inflammatory diseases. Studies show that the cell phenotypes of pyroptosis are manifested in osteoporosis, although the connection between osteoporosis and pyroptosis has not been definitely reported until now. Therefore, this study aims to illuminate the action of pyroptosis on osteoporosis and propose it as a novel pathogenesis of osteoporosis.

## Pyroptosis

Pyroptosis was first described in 1992 by Zychlinsky and colleagues (21) and is defined by the Nomenclature Committee on Cell Death (NCCD) as a programmed death mediated by gasdermin, which causes cells to swell until the cell membrane ruptures, causing the release of cell contents and activating a strong inflammatory response (22). Pyroptosis can be initiated by caspase-1 or caspase-11 and caspase-1 can be activated by a variety of inflammasomes, which are multi-protein signaling complexes. Once activated, inflammasomes assemble to form an intracellular macromolecular protein complex and convert the caspase-1 precursor to the active form (20, 23). Caspase-1 cleaves gasdermin D into the hydrophilic GSDMD-C-terminal domain and lipophilic GSDMD-N-terminal domain (24). The N-terminal domain is anchored on the cell membrane and polymerizes to compose a hollow annular oligomer at 10−20 nm in diameter (25–27), which disrupts the regular permeability barrier of the plasma membrane *via* breaking the concentration gradient of sodium and potassium, leading to an osmotic pressure change. When the number of pores exceeds the compensatory capacity of cells, water flows into the cells *via* an osmotic gradient, and then the cells swell, rupture, and die as a result (28). Furthermore, during this process, the IL-1β and IL-18 precursors are also cleaved into the mature form by caspase-1 (29, 30) and released through the gasdermin D pores or later during membrane rupture (31, 32). Moreover, additional immune cells will be recruited during this process to trigger an inflammatory response, which in turn induces pyroptosis (28).

**Figure 1:**
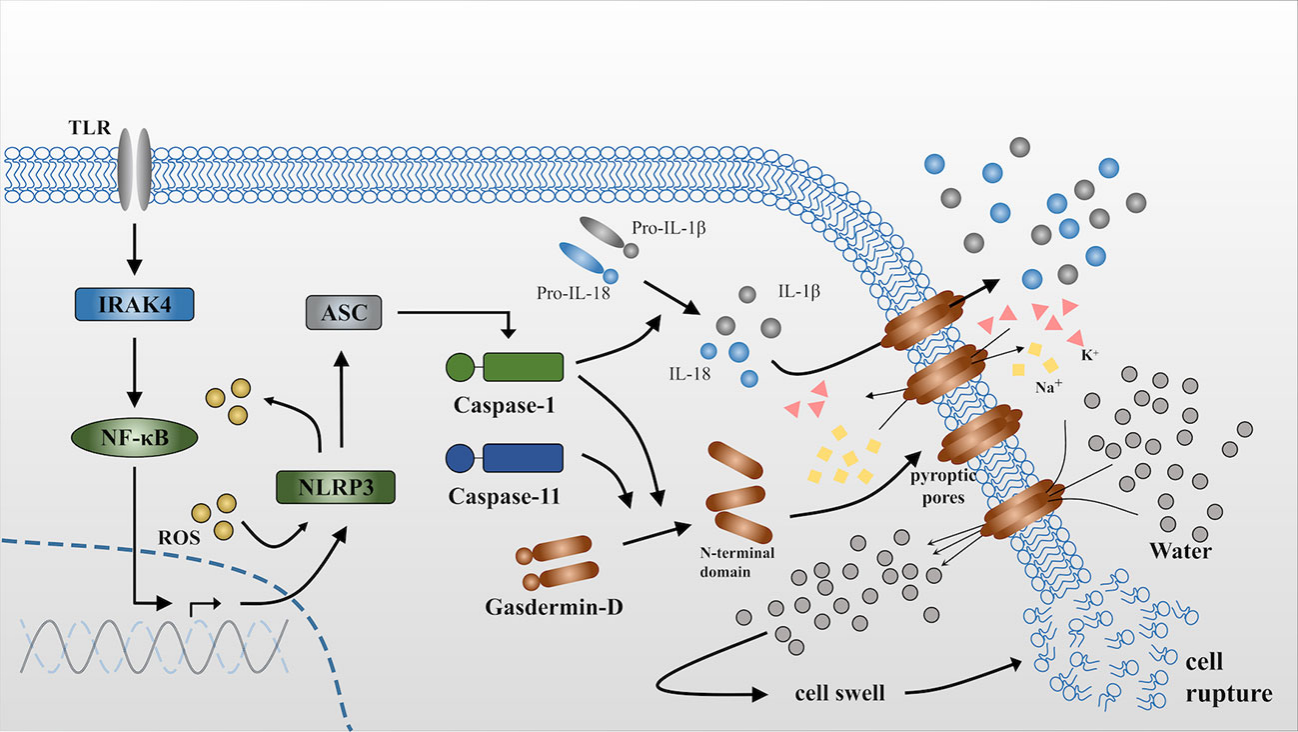
Release of IL-1β and IL-18 during pyroptosis. NLRP3 is activated *via* the NFκB-pathway when it receives stimulus from toll-like receptors (TLRs) (30). ASC is a bipartite molecule that bridges NLRP3 and caspase-1 precursors (41) and subsequently converts pro-caspase-1 into the activated form. Active caspase-1 can directly act on the precursors of IL-1β and IL-18 and cleave them into the mature form. By contrast, caspase-11 is itself the sensor for cytosolic lipopolysaccharide (LPS). Either caspase-1 or caspase-11 independently cleaves gasdermin D to produce N-terminal domains that anchor to the cell membrane and form pyroptotic pores. The influx of K^+^ and efflux of Na^+^ through the pyroptotic pores breaks the osmotic gradient of the cell and consequently, large quantities of water flow into the cell, resulting in cell swelling and rupture. IL-1β and IL-18 are released into the extracellular matrix through pyroptotic pores or accompanied by cell rupture. ROS can accelerate this process *via* triggering NLRP3 inflammasome formation as well as serving as effector molecules that will be demonstrated in the following.

In addition to caspase-1, caspase-11 can also mediate pyroptosis *via* the noncanonical pathway, it may directly recognize and bind with cytosolic lipopolysaccharide (LPS) with the priming of IFN-γ to initiate pyroptosis (33–37). Caspase-11 also acts on gasdermin D independently and produces an N-terminal fragment that oligomerizes and combines with the cell membrane to form pyroptotic pores (31, 38). However, Nobuhiko Kayagaki studied *Casp11* gene-targeted mice and *Casp11* ^-/-^ mice and showed that caspase-11 is incapable of directly cleaving the IL-1 and IL-18 precursors, although is an essential effector of non-canonical caspase-1 activation and secretion of mature IL-1β and IL-18 (39, 40) (**Figure 1** (30, 41)).

Pyroptosis is a classical inflammatory reaction that induces T lymphocyte and macrophage activation. Nevertheless, excessive pyroptosis can lead to high levels of cell death, tissue injury, and organ failure, and even cause autoimmune inflammation or septic shock, resulting in irreversible damage to the body (42, 43). Numerous research has indicated that pyroptosis is closely connected with other diseases that cover almost all organs or tissues in the human body. For instance, pyroptosis is thought to be closely linked to various bacterial, viral, and fungal infections (44–46). Pyroptosis is also of great importance in the pathogenesis of many cardiovascular diseases, like atherosclerosis with the activation of NLRP3 (47, 48). Furthermore, Tan MS et al. reported that cerebral NLRP1 levels both *in vitro* and *in vivo* were upregulated, indicating the important role of NLRP1/caspase-1 signaling in the progression of Alzheimer’s disease (49). In addition, an array of evidence also suggests that pyroptosis performs a major part in the development of metabolic diseases and spontaneous inflammatory diseases (28). Moreover, IL-1 and IL-18 are undoubtedly important nodes of pyroptosis while NLRP3 is a key effector of pyroptosis.

Except for the diseases presented above, osteoporosis has not been definitely and directly connected with pyroptosis, although both phenomena have a close relationship with inflammation. Indeed, pyroptosis participates in the pathogenesis of osteoporosis mainly *via* activating NLRP3 and secreting mature IL-1β and IL-18, which will be demonstrated as follows.

## IL-1

As mentioned above, T lymphocytes and macrophages are activated during pyroptosis. The activation of T cells and macrophages can induce the release of cytokines, including IL-1, IL-18, interferon-γ (IFN-γ), and TNF-α, etc. (50–52). Moreover, IL-1β is secreted out of the cell during pyroptosis. Therefore, a considerable elevation of IL-1 occurs during the process of pyroptosis, which upregulates osteoclastic formation in several ways. For example, early in 1989, Boyce BF et al. injected IL-1α once daily for three days into normal mice and indicated that IL-1 can stimulate bone formation and absorption systemically and that it has a profound long-term effect on bone remodeling (53). Later in 2007, it was demonstrated by Zwerina J et al. that IL-1 induces the expression of RANKL in mesenchymal stem cells (MSCs) and directly affects osteoclasts by enhancing the RANK expression. Further study by Aoki et al. in 2015 demonstrated that the overexpressed human IL-1α gene can cause osteopenia in hIL-1α Tg mice (54).

### Effects of IL-1β on Bone Metabolism

There are 11 members in the IL-1 ligand family, and IL-1β has been regarded as the major therapeutic target for a variety of inflammatory conditions (55, 56). IL-1β binds to its receptor and initiates IL-1 signal transduction, which includes mitogen-activated protein kinase (MAPK) cascade and the NFκB-pathway (57, 58). These signaling pathways cause the activation of some transcription factors, such as c-Jun N-terminal kinase (JNK), AP-1, NF-κB, and p38 MAPK (55, 59).

It was first reported by S-M Dai et al. in 2004 that IL-1 stimulates osteoclastogenesis indirectly through the upregulation of the production of RANKL from rheumatoid arthritis synovial T cells (60). Binding IL-1β with its receptors on T lymphocytes, B lymphocytes, and macrophages promotes the production of RANKL (61), which combines with the RANK of osteoclast precursor cells and contributes to the differentiation and activation of osteoclasts (62, 63). For example, it was reported by Lee SK in 2006 that endogenous IL-1 enhances the response to RANKL of bone marrow cells, and this effect seems to be mediated by mechanisms associated with the enhancement of JNK activity (64). Moreover, IL-1β can activate osteoclasts by increasing the production of M-CSF (macrophage colony-stimulating factor) and also inhibit osteoclast apoptosis (65).

Furthermore, IL-1 is also regarded as a crucial mediator of TNF-mediated osteogenesis (66, 67). TNF and IL-1 are initially involved in different signaling pathways, which are fused with the activation of NF-κB and the stimulation of the MAPK system. Therefore, the crosstalk effect of the two cytokines provides an effective signal to osteoclastogenesis, inhibits osteoblastic function, and regulates the lifespan of skeletal cells (19). For example, Shi Wei et al. reported that IL-1 regulates the osteoclastogenic effect of TNF-α by directly stimulating the differentiation of osteoclast precursors and enhancing the RANKL expression of stromal cell (68). Later in 2007, Zwerina J et al. crossed arthritic human TNF-transgenic (hTNF-tg) mice with IL-1α and β-deficient (IL-1^-/-^) mice and observed that compared with hTNFtg mice, osteoclast formation and bone erosion were greatly reduced in IL-1^-/-^hTNFtg mice, suggesting that IL-1 must be an indispensable mediator of TNF-induced osteoclastic formation, and this finding was further reinforced by K Polzer et al. with similar methods in 2015 (69). Moreover, several researchers have proved that TNF-α and IL-1β have a potent anti-apoptotic effect in osteoclasts. In 1999, Eijiro Jimi et al. confirmed that IL-1 induced the viability and multinucleation of osteoclasts and stimulated their pit-forming activity due to the ability to activate NF-κB (70). The prolongation of the osteoclastic lifespan may represent a crucial contribution toward accelerated bone resorption (19, 71).

On the other hand, IL-1 also has an impact on osteoblasts. IL-1α can induce the viability of decreased MC3T3-E1 cells and inhibit osteoblast differentiation through the JNK and p38 MAPK pathways (72). Zhang YZ et al. performed the knockdown of TLR4 in osteoblasts and reduced the levels of IL-1, TNF-α, and IL-6, and subsequently, the cell viability improved because of the inhibition of the inflammatory pathway (73). Conversely, in an osteoporosis model, rats subjected to an ovariectomy showed remarkably higher levels of IL-1, IL-6, and TNF-α in serum compared with control rats and the differentiation of MSCs was blocked as well (74). Obviously, osteoblasts had lost their abilities in an inflammatory environment.

### Effects of IL-1β on Dendritic Cells

Immune cells may have the same ability to differentiate into osteoclasts. Dendritic cells (DCs), which are regarded as professional antigen-presenting cells capable of efficiently activating naive T lymphocytes, are able to differentiate into osteoclasts *in vitro* at the early development stage (75). Indeed, immature dendritic cells can be induced into osteoclasts with the assistance of M-CSF and RANKL (76). DCs contribute to osteoclastogenesis indirectly and their major ability is activating naive T cells, which can then produce RANKL and stimulate osteoclasts differentiation (77, 78). Interestingly, it was suggested by Akagawa that DC-derived osteoclastic formation is dramatically faster and more efficient in terms of fusion rate than the classically described monocyte-derived osteoclastic formation pathway (79). The notion of the transdifferentiation of DCs was further reinforced in 2007 by Carole Speziani, who showed that murine IL-1β and TNF-α can increase bone resorption and DC fusion into osteoclasts when M-CSF and RANKL are present and compared with TNF-α, IL-1β had a higher efficiency in promoting bone resorption (80).

## IL-18

As an IL-1 cytokine superfamily member, IL-18 is also secreted out of the cell *via* the processing of caspase-1 (28, 56). After combining with its receptor, IL-18 shares signaling pathways with IL-1 receptors and toll-like receptors (TLRs) and activates many transcription factors, like NF-κB, AP-1, and MAPK (81, 82). In 1999, Gracie et al. showed that the mRNA and protein of IL-18 are abundant in the joints of RA patients and proved that IL-18 can stimulate the significant production of IFN-γ, GM-CSF, and TNF-α (83). Indeed, IL-18 plays a key role in the increased production of TNF-α and IL-1β (84). For example, Joosten LA et al. reported that the significant suppression of IL-1β and TNF-α levels emerged after the blockade of endogenous IL-18. The role of IL-1β and TNF-α in osteoclastic formation has been described above. Therefore, IL-18 can facilitate the differentiation of osteoclasts *via* several pathways, including inducing the secretion of interferon -γ (IFN-γ) as well as acting on T lymphocytes.

### IL-18 Induces the Secretion of IFN-γ

IL-18 is characterized as an IFN-γ inducing factor because of its ability to augment the production of IFN-γ by natural killer cells and activated T cells in the presence of IL-12 (85, 86). For example, primary data derived from gene knockout experiments by Yoshimoto T show that IL-18 might favor the Th1 response and subsequently induce IFN-γ production *in vivo* (87). IL-18 from CD8α+ dendritic cells can stimulate the IFN-γ secretion of memory CD4+ T cells in the spleen which activate the NLRC4 inflammasome against S. Typhimurium (88).

Initially, IFN-γ was described as the anti-osteoclastogenic factor because of its potent inhibitory effect for osteoclastogenesis *in vitro* (51). This notion reinforced that IFN-γ is a bone resorption inhibitor when Vermeire K et al. found that silencing of IFN-γR^–/–^ signaling would result in a severer collagen-induced arthritis and faster bone resorption (89). However, it is also observed that IFN-γ can promote bone resorption and lead to bone loss in various conditions. For example, it was demonstrated by Baker PJ that a lack of IFN-γ in mice led to decreased bone loss (90). Furthermore, as a potent antigen presentation inducer, IFN-γ is also an inducer of T cell activation. When IFN-γ levels are increased *in vivo*, activated T cells secrete pro-osteoclastogenic factors, offsetting the anti-osteoclastogenic influence of IFN-γ (19). The controversy was eventually solved later in 2007 by Yuhao Gao et al., who showed that IFN-γ had both indirect pro-osteoclastogenic and direct anti-osteoclastogenic effects *in vivo* while the net balance of the two opposing forces is biased towards bone resorption under inflammatory conditions (91). Therefore, IL-18 promotes osteoclastogenesis *via* increasing the production of IFN-γ under the inflammatory condition caused by pyroptosis.

### IL-18 Acts on T Lymphocytes

**Figure 2:**
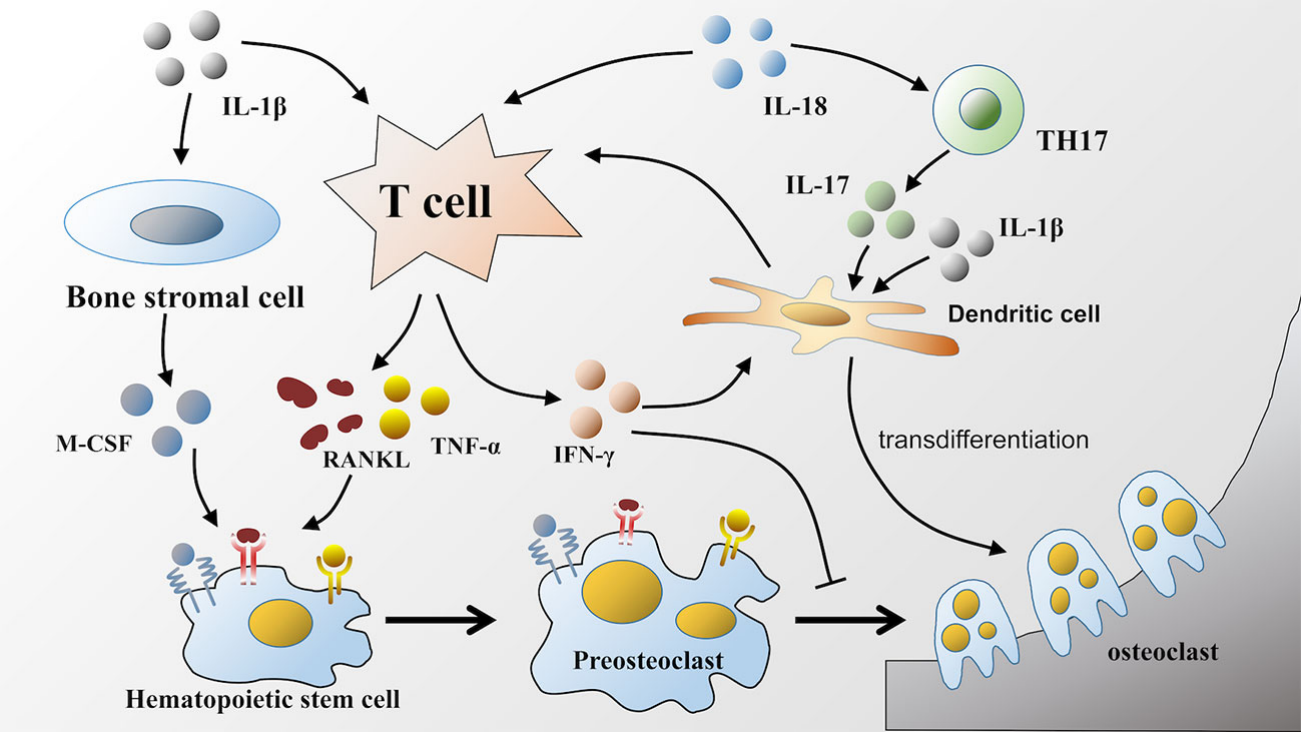
Effects of IL-1β and IL-18 on osteoclastic formation. IL-1β and IL-18 released during pyroptosis facilitate osteoclastic formation in various ways. IL-1β and IL-18 can both act on T lymphocytes to upregulate the production of RANKL. Furthermore, IL-1β also participates in TNF-α mediated osteoclastogenesis, as well as facilitates the production of M-CSF by bone stromal cells. RANKL, TNF-α, and M-CSF bind to corresponding receptors respectively on hematopoietic stem cells to promote its differentiation into osteoclasts. Unlike IL-1β, IL-18 can increase the production of IFN-γ by T lymphocytes, which has dual effects on osteoclastic differentiation. Moreover, IL-18 can also upregulate the production of IL-17. Either IL-17 or IL-1β can induce the transdifferentiation of dendritic cells into osteoclasts.

IL-18 can also directly act on T lymphocytes and subsequently facilitate bone resorption. For example, an observation by S-M Dai showed that IL-18 indirectly stimulated osteoclastic formation through the upregulation of the production of RANKL from T cells, which is as effective as IL-1β but less potent than TNF-α (60). Moreover, IL-18 is also involved in driving the response of TH17 cells to induce IL-17 production (92, 93), and IL-17 can directly induce human monocytes to differentiate into osteoclasts in the presence of TNF-α and RANKL (94). Similar to IL-1β, IL-17 can also increase murine DC development into osteoclasts, which likely occurs *via* the induction of IL-1β or TNF-α (80) (**Figure 2**).

## NLRP3

Inflammatory responses must be induced in macrophages before inflammasomes are activated, and chronic inflammatory responses represent a primary risk factor for inflammatory or autoimmune diseases (95). As demonstrated above, IL-1β and IL-18 are known to produce a number of effects in osteoclastogenesis; therefore, excessive activation of NLRP3 can affect osteoclastic formation by processing IL-1β and IL-18 precursors. Moreover, there are two signals required for the activation of the NLRP3 inflammasome: the NF-κB-dependent pathway and an agonist to induce oligomerization (96). The NF-κB-dependent pathway is of great significance in osteoclastic differentiation. Therefore, excessive activation of inflammasomes would play a pivotal role in the occurrence and development of inflammatory/autoimmune diseases.

### Effects of NLRP3 on the Release of IL-1β and IL-18

NLRP3 is capable of promoting progressive and debilitating arthritis characterized by granulocytic infiltration, elevated cytokines, bone erosion, and osteoporosis in humanized mice. For example, John N. Snouwaert et al. demonstrated that IL-1β was elevated in the samples from NLRP3 humanized animals but IL-18 and IL-6 levels were significantly higher. Moreover, kyphosis as well as imageological changes to the hindlimbs were observed in mutant mice with advancing age, which is consistent with osteoporosis (97). Moreover, *Jenko B* et al. showed that at the genetic level, the NLRP3 inflammasome components expressed were significantly higher in patients with RA compared to controls and the NF-κB signaling pathway was upregulated as a result, which also facilitates the formation of osteoclasts (98, 99). Another study also investigated the regulation of NLRP3 inflammasomes in the release of IL-1β. Vande Walle et al. indicated that the rheumatoid arthritis susceptibility gene A20 was lacking, increasing the expression of pro-IL-1β and NLRP3 genes, which led to caspase-1 activation mediated by the NLRP3 inflammasome, IL-1β secretion, and the occurrence of pyroptosis, although a deficiency of NLRP3 greatly suppressed this process (100).

### Interplay of NLRP3 With ROS

ROS are produced by some known NLRP3 inflammasome activators, and they are critical triggers for the formation and activation of the NLRP3 inflammasome (101). In 2007, Cruz CM et al. discovered that inhibiting NADPH oxidase-derived ROS can prevent the activation of caspase-1 induced by ATP and the secretion of IL-1β in alveolar macrophages (102), and they initially proposed the hypothesis of ROS involvement in the formation and activation of the NLRP3 inflammasome. In the following year, this hypothesis was further substantiated by Dostert C et al., who significantly knocked down the p22^phox^ subunit of NADPH oxidase and found that the release of IL-1β was significantly suppressed (103). However, apart from serving as a triggering factor to activate NLRP3, ROS have also been demonstrated to be effector molecules that result in pathological processes (104). When NLRP3 inflammasomes are activated by various stimuli, the local inflammatory response occurs with the production of ROS and cytokines (105). If ROS derived from NLRP3 activation are significantly generated relative to eliminated during this process, then intracellular and extracellular oxidative stress occurs and subsequently contributes to an array of pathophysiological processes and respective diseases. For example, recent studies have also indicated that ROS are important components in the pathogenesis of osteoporosis *via* upregulating the differentiation of osteoclasts and facilitating the resorption of bone (106, 107). Therefore, as is demonstrated at the beginning of this review, excessive production of ROS due to the aging process activates NLRP3 inflammasomes, which in turn produce more ROS and aggravate the condition of high amounts of ROS. Under the circumstances, excessive bone resorption emerges due to the overactivation of osteoclasts by ROS, which eventually results in osteoporosis.

### Effects of NLRP3 on Osteogenesis

NLRP3 directly inhibits osteogenesis by affecting osteoblasts and plays a core role in osteoporosis induced by inflammation. Early in 2008, McCall SH et al. proved that osteoblasts expressed NLRP3 which mediated bacterially induced cell death and participated in bone loss during inflammation (103). NLRP3 is considered essential in many bacterial infections caused by osteoporosis (100, 108–110). When NLRP3 inflammasomes increase, the caspase-1 pathway is activated and the expression levels of IL-1 and IL-18 are upregulated, resulting in the death of osteoblasts (111). Although not explicitly stated, this finding is evidence of pyroptosis in osteoblasts. In addition, in estrogen-deficient osteoporosis, direct bacterial infection was not observed but NLRP3 levels still rose, the viability of osteoblasts was significantly increased, and osteoporosis was also relieved when NLRP3 was inhibited or inactivated (112, 113).

## Conclusions and Perspectives

**Figure 3:**
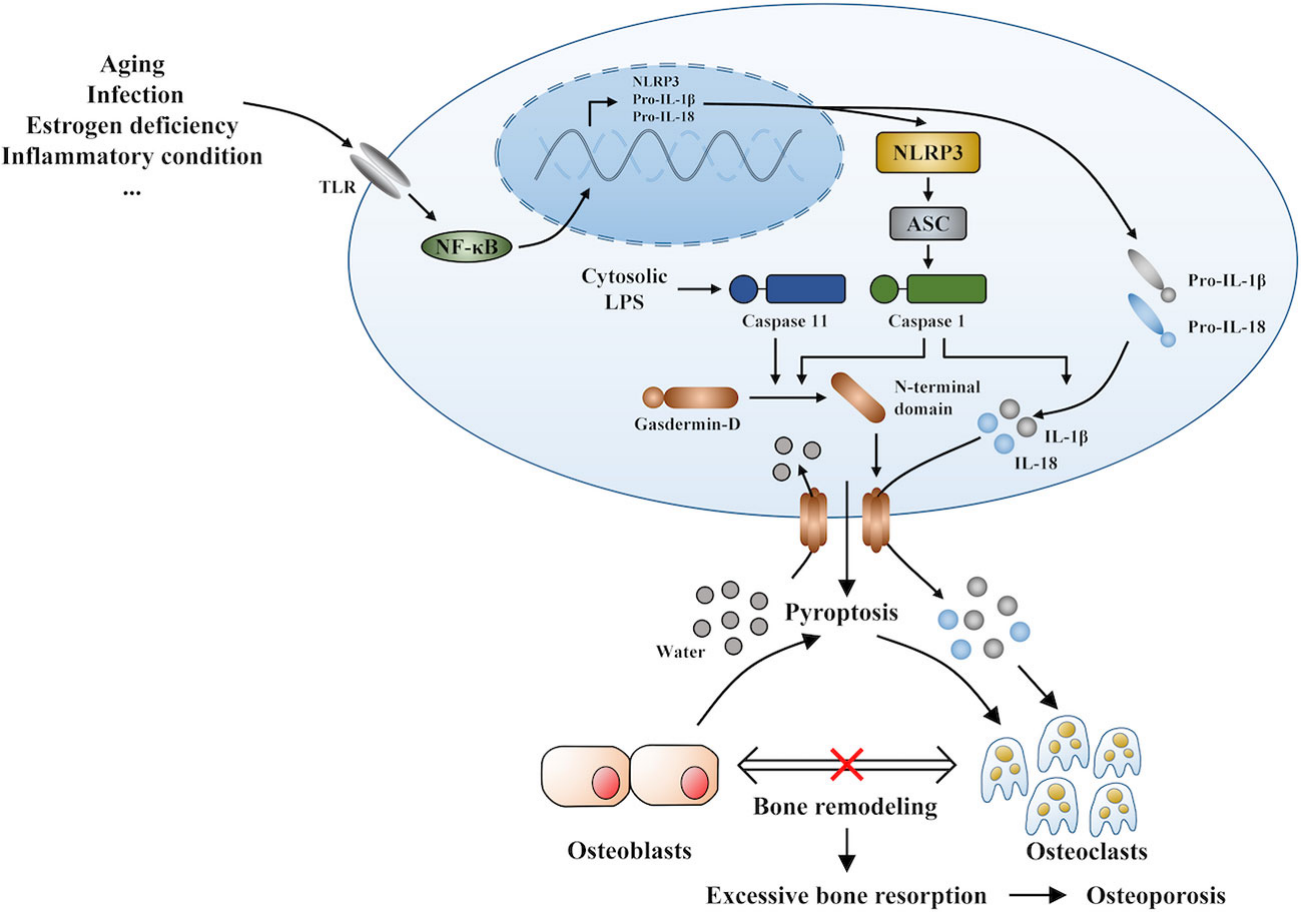
The novel hypothesis of pyroptosis as an osteoporosis pathogenesis.

**Figure 4:**
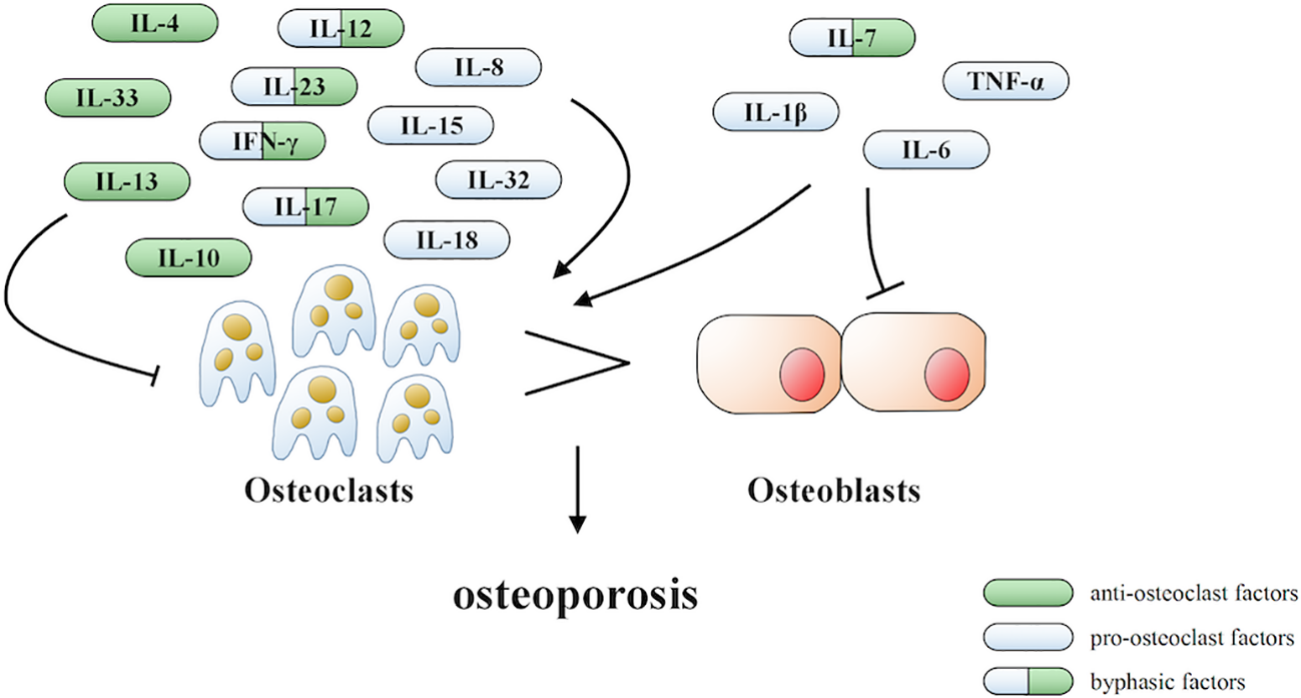
Cytokines involved in the pathogenesis of inflammation-induced osteoporosis.

Under inflammatory conditions, a series of corresponding molecules recognized by inflammasomes activate caspase-1 and cleave gasdermin D into a reactive N-terminal fragment with the participation of caspase-11. The N-terminal fragments anchor to the cell membrane and lead to cell swelling and death *via* the formation of pyroptotic pores. Precursors of IL-1β and IL-18 are processed by caspase-1 during pyroptosis and secreted into the extracellular milieu along with cell debris, which aggravates the inflammatory condition. IL-1β and IL-18 can directly or indirectly facilitate the differentiation of hematopoietic stem cells and preosteoclasts into mature osteoclasts, which leads to the excessive formation of osteoclasts. The formation of osteoclasts exceeds that of osteoblasts, which leads to superfluous bone resorption and an imbalance of bone remodeling, which subsequently results in osteoporosis. Moreover, the stimulation of pyroptosis on osteoclastogenesis may be more obvious in age-related osteoporosis due to the dual action of ROS as a triggering factor and an effector of NLRP3 inflammasomes. Therefore, we speculate that pyroptosis is the main pathogenesis of osteoporosis (detailed hypothesis in **Figure 3**). When the body is exposed to external stimuli, such as bacterial infection, estrogen deficiency, or aging signals, many inflammatory pathways are activated, IL-1 and IL-18 secretion of inflammatory factors increase, and NLRP3 inflammasomes amplify this signal. Pyroptosis occurs in osteoblasts, which further activate osteoclasts and leads to the aggravation of bone loss. The current clinical drugs used to treat osteoporosis are mainly classic anti-osteoclast drugs, like bisphosphonates or expensive osteogenic teriparatide. Besides, denosumab and romosozumab show promise in the treatment of osteoporosis according to recent research (114). Our study highlights the potential of essential components during pyroptosis, such as NLRP3, caspase-1, or the N-terminal of gasdermin D, as drug targets. However, the detailed activation and action mechanisms are not thoroughly understood, which has hampered the discovery and development of novel therapeutics against these targets. Here, we summarized some important factors involved in pyroptosis and their crosstalk pathways related to osteoporosis (**Table 1**) (108, 109, 113, 115–118) and some cytokines involved in the pathogenesis of inflammation-induced osteoporosis (**Figure 4**). Some of the pro-inflammatory cytokines, such as IL-7, IL-12, IL-23, and IL-17, as well as INF-γ, have been shown to possess dual, osteoclastogenic, and anti-osteoclastogenic properties. It seems that their net effect depends on the specific pathophysiological condition of the bone in which they are being studied in *in vivo*, while in experiments *in vitro* it depends on the developmental stage of osteoclasts. Among them, IFN-γ can suppress osteoclastogenesis *via* directly targeting osteoclast precursors, but indirectly promote osteoclast formation and bone resorption by stimulating antigen-dependent T cell activation and production of the osteoclastogenic factors RANKL and TNF-γ. Under the conditions of estrogen deficiency, infection, and inflammation, the net effect of these two opposing properties is biased toward bone resorption (91). And the dual effects of IL-12 can be regarded as the opposing properties of IFN-γ because it serves as a potent inducer of IFN-γ (119). The effect of IL17 is concentration-dependent, which means low concentrations of IL-17 can promote bone resorption while high concentrations of IL-17 can restrain the differentiation of osteoclasts (120, 121). Hence, further investigations into the action of additional components present in pyroptosis on the activation and proliferation of osteoclasts and osteoblasts are of great significance in the pathogenesis of osteoporosis as well as the development of therapeutic strategies.

**Table 1:** Some important factors involved in pyroptosis and their crosstalk pathways.

	Effects	Crosstalk pathways	Reference
IL-1	Recruit inflammatory cells to agglomerate and expand inflammatory response	NLRP3 mRNA/NLRP3/IL-1β pathway MAPK/p38/Smad pathway JNK/NF-κB pathway	*Elshaier AM et al.* (114)
IL-18	Recruit inflammatory cells to agglomerate and expand inflammatory response	TLR4/MyD88/NF-κB pathway	*Liu DW et al.* (115)
NLRP3	Identify pathogen-associated molecular patterns (PAMPs) and danger-associated molecular patterns (DAMPs), recruit and activate caspase-1, promote the maturation and secretion of multiple pro-inflammatory cytokines	NLRP3/Caspase-1/IL-1β(IL-18) pathway Wnt/β-catenin pathway ROS/MAPKs/NF-κB/NLRP3 pathway	*Xu LJ et al.* (113) *Yoshida et al. *(108) *An YN et al.* (116)
Gasdermin-D	Induce cell membrane perforation, cell rupture, release of contents, cause inflammatory response	Caspase-1/GSDMD pathway Caspase-4,5,11/GSDMD pathway	*Ran S et al.* (109)
Caspase-1	Cut the precursors of Gasdermin D, IL-1β and IL-18	Caspase-1/GSDMD pathway NLRP3/Caspase-1/IL-1β(IL-18) pathway	*Xu LJ et al.* (113) *Ran S et al.* (109)
TLR	Receiving, identifying and transmitting signals	TLR4/JNK/NF-κB pathway Wnt/β-catenin pathway	*Pei JP et al.* (117)

## Author Contributions

ZT and JW made substantial contributions to the conception and design of the work. RY, WD, SZ, YM, SQ, and KY performed the acquisition and analysis of literature. ZT, JW, and KW drafted the work. All the authors approved the version to be published. LT and YZ are responsible for this study. All authors contributed to the article and approved the submitted version.

## Conflict of Interest

The authors declare that the research was conducted in the absence of any commercial or financial relationships that could be construed as a potential conflict of interest.

## Abbreviations

IL-1, interleukin-1; IL-18, interleukin-18; NLRP3, nucleotide-binding domain, leucine-rich-containing family, pyrin domain containing 3; GSDMD, gasdermin D; ROS, reactive oxygen species; DC, dendritic cells; RANK, receptor activator of NF-κB; RANKL, receptor activator of NF-κB ligands; M-CSF, macrophage colony-stimulating factor; MSCs, mesenchymal stem cells; RA, rheumatoid arthritis.
